# Challenges in the abortion supply chain: a call to action for evaluation research

**DOI:** 10.1186/s12978-020-01060-0

**Published:** 2021-01-20

**Authors:** Ghazaleh Samandari, Nathalie Kapp, Christopher Hamon, Allison Campbell

**Affiliations:** 1 Ipas, P.O Box 9990, Chapel Hill, NC 27701 USA; 2 University of North Carolina, Chapel Hill, USA

**Keywords:** Abortion, Supply chain management, Logistics, Safe abortion, Abortion supply, Review

## Abstract

Reducing the burden of unsafe abortion rests considerably on women’s ability to access appropriate and timely treatment or services. A critical component of that care relies on a functional supply chain to ensure availability of abortion drugs and supplies within the health system. Disruptions in the supply of medical abortion drugs delay provision of abortion services and can increase the risks to a woman’s health. We examine the ways in which supply chain management (SCM) affects women’s ability to access safe and timely abortion to meet their reproductive health needs and highlight the gap in evaluation research on which SCM interventions best improve access to safe abortion care.

SCM comprises a critical component of efficient and sustainable abortion service provision and is a requisite for expansion of services. Furthermore, governments are responsible for safeguarding links in the abortion supply chain, from registration to distribution of abortion drugs and supplies. Strategic public–private partnerships and use of innovative local or community-based distribution mechanisms can strengthen supply chain systems. Finally, alternatives to the pull-based models of distribution could alleviate bottlenecks in the final steps of abortion supply chains. Programs aimed at increasing access to safe and comprehensive abortion care must include SCM as a foundational component of service provision. Without access to a sustainable and affordable supply of abortion drugs and equipment, any attempt at providing abortion services will be critically limited. More implementation research is needed to identify the most effective interventions for improving SCM.

## Introduction

Unsafe abortion accounts for 7 million annual cases of abortion-related complications and accounts for 8% of maternal deaths worldwide [1]. While abortion is restricted in many settings around the world, only 6% of women live in countries where there are no legal indications for the procedure [2, 3]. As such, reducing the burden of unsafe abortion rests considerably on women’s ability to access appropriate and timely treatment or services. A critical component of that care relies on a functional supply chain to ensure availability of abortion drugs and supplies within the health system. It is particularly important that abortion drugs and supplies be readily available at the point of care in settings where there are gestational age limits on legal abortion or where women delay seeking abortion care due to stigma or other barriers. Disruptions in the supply of medical abortion drugs such a misoprostol or mifepristone, manual vacuum aspiration (MVA) kits or other key medicines such as analgesics and antibiotics delay provision of abortion services and can increase the risks to a woman’s health [4].

Studies of supply chain management (SCM) as relating to contraceptive security have demonstrated its impact on women’s ability to utilize modern contraception to meet their reproductive health needs [5, 6]. Inefficiencies such as weak management systems, poor funding for procurement and distribution, lack of trained staff and governmental policies that restrict acquisition or delivery of supplies create significant bottlenecks in the supply chain [5, 7, 8]. Bottlenecks contribute to routine stock-outs of contraceptive commodities within national health systems, greatly limiting women’s access to desired contraceptive care [9–12]. In addition to addressing failures along the supply chain, these studies suggest alternative models of distribution and private–public partnerships as solutions to improving SCM for contraceptive supplies [13].

There is a current lack of research on how supply chain management impacts abortion services. Although needed commodities overlap significantly with maternal health programs, abortion care may be overlooked or ignored in government’s planning for peripartum supplies. Intervention studies in this domain are lacking, yet some descriptive studies suggest that proper management of the abortion supply chain facilitates provision of timely, necessary and life-saving abortion services, while poor management at best delays services and at worst results in unsafe abortion practices. We examine the ways in which SCM affects women’s ability to access safe and timely abortion care to meet their reproductive health needs.

## Main body

### SCM is essential for abortion service sustainability and expansion

Direct, small-scale abortion supply provision can result in critical shortages once program funding ends. Providers in Malawi and Cameroon faced significant declines in MVA kit availability following initial training or piloting of interventions due to a lack of follow-up funding and capacity for supply management, forcing a reversion to use of dilation and curettage, a practice associated with greater rates of maternal morbidity and pain [14, 15]. In some contexts, providers report re-using MVA supplies well past what is recommended, with unknown safety implications. As such, initial gains in MVA training and services, particularly at the lowest levels of the health system often diminish due to a lack of affordable MVA kits [16]. A struggling supply chain not only affects sustainability of abortion services but can also limit the capacity of programs to expand their services. Abortion providers in China and Vietnam both identified issues with drug and equipment supplies as one of the main barriers to expansion of medical abortion services [17, 18]. In Guatemala and Kyrgyzstan, the success of medical abortion and postabortion care (PAC) training and service delivery programs depended heavily on continued availability of MVA kits and strengthening of procurement and distribution systems to prevent MA drug stock-outs [19, 20].

### Strong government commitment to abortion SCM undergirds every link in the supply chain

From initial registration of abortion drugs to price negotiation for affordable procurement and maintenance of distribution channels, governments are the lynchpin in effective abortion SCM initiatives [23]. Governments play an important role in identifying appropriate manufacturers of misoprostol, negotiating beneficial procurement terms with manufacturers and creating oversight mechanisms for quality assurance which includes specific government budget line items for quality assurance testing. An assessment of the introduction of medical abortion in Vietnam showed that the single-most urgent action needed from the government for sustainable abortion services, after registering mifepristone in 2003, was to ensure a steady, affordable supply of abortion drugs, which included negotiating lower prices for medical abortion drugs both with local producers and international distributors [24]. The Ghanaian government’s initiative to add MVA kits to the national List of Essential Medicines & Supplies contributed significantly to their MVA scale-up effort [16]. On the other hand, the government policy of administrative approval for all commodity purchases in public facilities across Ghana created a cumbersome procurement process that limits efficient acquisition of abortion supplies [16]. Particularly due to their mandate for oversight of medical drugs and supplies, governments are uniquely positioned to ensure efficiency in the abortion supply chain system.

### Strategic partnerships are crucial to a functional abortion supply chain system

Non-governmental organizations (NGOs) and private sector partners play an important role in abortion supply management, especially in settings where legal indications for abortion are restricted. Partnerships between private and public sectors or NGOs and governments, can be effective in registration, procurement and distribution of misoprostol [21]. Private–public coordination has also been leveraged to increase access to abortion equipment, such as in the partnership between the Ghanaian government and a consortium of five NGOs who contributed to scaling-up MVA kit distribution mechanisms to midwives serving in rural areas [16]. In settings with medicines registered for abortion, distribution of drugs can also be increased by partnering with direct-to-consumer outlets such as pharmacists or community health workers [22].

### Community-based distribution can supplement existing supply chain mechanisms

A study of community-based advocacy for misoprostol in Tanzania and Kenya demonstrated the significance of local strategies for innovative misoprostol delivery [22]. In Kenya, a local NGO worked with traditional birth attendants (TBAs) to expand access to misoprostol through a taxi-based on-demand delivery service in communities where pharmacists did not stock the drug. When local pharmacies did not supply misoprostol in Tanzania, a local organization trained their own staff to provide abortion counseling and support and provided drugs directly to women through their own makeshift pharmacy (an alliance with local doctors ensured women’s safety in the event of complications) [22]. A number of programs aimed at preventing postpartum hemorrhage have successfully utilized community-based distribution of misoprostol. Effective implementation of such programs is facilitated by supportive policies, guidelines and formal planning for drug supply chain management and can offer a model for similar community-based distribution of medical abortion drugs [25].

### Alternative models of distribution improve SCM for abortion drugs and equipment

Traditional pull-based distribution models—which rely on complex and time intensive systems of stock record-keeping and procurement requests—may be exacerbated further in the context of abortion drugs and supplies where off-label misoprostol use, clandestine service provision or consumption underreporting due to stigma may occur [25]. Daff et al. devised an alternative “informed push model” of contraceptive commodity management in Senegal that engages dedicated logisticians at the district level to maintain up-to-date stock records, sell commodities on a consignment basis, and stock at least two months of supplies per facility at all times. Although initially time and resource-intensive, the development of a sustainable distribution system at the local level led to drastic reductions in contraceptive commodity stock-outs in Senegal [11]. At the very least, programs that work to increase access to safe abortion should include supply system strengthening as part of their intervention.

### There is a lack of sufficient evaluation of abortion SCM

SCM is a critical component of abortion service programming, yet no current studies evaluate the impact of abortion SCM programming on abortion access. The lack of any peer-reviewed or grey literature articles testing interventions to improve abortion SCM, despite its importance in delivering quality abortion care, demonstrates a significant dearth of attention on abortion SCM in the reproductive health and rights field. Providers repeatedly point to supply chain barriers as one of the primary challenges to abortion service provision, yet few abortion service training programs directly address SCM. Abortion supplies may be subject to additional legal and social barriers that limit their availability within traditional supply chain systems, further highlighting the importance of additional research in this area. Furthermore, none of these programs evaluate the unique challenges of ensuring a robust supply chain for quality abortion drugs and equipment. Evidence from such studies could greatly facilitate the planning and development of effective SCM interventions for abortion service delivery programs.

## Conclusion

Timely access to affordable abortion drugs and supplies forms the foundation upon which provision and expansion of abortion services rest. Although some abortion commodities may overlap with maternal child health programming, the parallel abortion services are more likely to be short-changed in the face of scarcity. Research is needed to identify interventions which meaningfully improve supply chain systems for abortion commodities as well as their effect on women’s health outcomes to fully understand the impact of SCM on safe abortion care. Such evidence could be used to rally governments and strategic partners around interventions to strengthen supply chain systems, and to advocate for inclusion of SCM interventions within abortion programming.

The onset of COVID-19 has exacerbated issues in global and local abortion supply chains, further limiting access to safe and timely abortion for millions around the world. Delays in production and export for distribution of abortion medication and supplies from counties which closed their borders during the pandemic highlight upstream issues in manufacturing that will require more widespread effort in our field to address. However, the pandemic has also encouraged significant innovation, particularly through telemedicine and mailing of abortion drugs, which has helped ensure access during the crisis. How we respond to emerging issues today may offer additional reproductive health choices, or may even expand access to some women and girls, moving forward beyond the pandemic.

## Data Availability

Not applicable.
